# Letter to Editor: Methodological constraints in assessing dietary and urinary molybdenum

**DOI:** 10.1002/jpr3.70195

**Published:** 2026-07-05

**Authors:** Nan Du, Jocelyn Silvester

**Affiliations:** ^1^ Division of Gastroenterology, Hepatology and Nutrition Boston Children's Hospital Boston Massachusetts USA; ^2^ Celiac Center, Division of Gastroenterology Beth Israel Deaconess Medical Center Boston Massachusetts USA

Dear Editor,

We thank Dr. Erika Sievers for their interest in our paper,^1^ and for providing insightful commentary^2^ on the importance of careful contextual documentation to interpret molybdenum (Mo) levels in children.

We agree that comprehensive analytical assessment of dietary Mo is both pertinent and essential for understanding and preventing excessive urinary Mo levels. Additionally, we concur that adopting a multidimensional approach, such as preserving dietary samples and assessing renal function, offers contextual understanding of Mo levels in biologic samples. Unfortunately, preservation of dietary samples is difficult for study participants to comply with (particularly for children), can be cost prohibitive, and Mo's bioavailability varies in different food matrixes.

In our study, our 3‐day dietary record was designed to focus primarily on dietary arsenic exposure. As such, other dietary sources of Mo (which is in legumes, vegetables, and animal organs)^3^ were not systematically recorded. Participants were prompted twice to include any additional foods not included in the food record. Notably, none of the participants in our study had documented kidney disease or known renal impairment. Similarly, as our primary aim was arsenic levels, we did not measure plasma metal concentrations which may have been a more helpful proxy for long‐term intake for Mo.^4^

Overall, understanding the various trace elements found in food, how to monitor them and how they interact are important questions that need to be addressed to advance our understanding of nutrition and health.

## CONFLICT OF INTEREST STATEMENT

The authors declare no conflicts of interest.
